# Combining Dietary Sulfur Amino Acid Restriction with Polyunsaturated Fatty Acid Intake in Humans: A Randomized Controlled Pilot Trial

**DOI:** 10.3390/nu10121822

**Published:** 2018-11-23

**Authors:** Thomas Olsen, Bente Øvrebø, Cheryl Turner, Nasser E. Bastani, Helga Refsum, Kathrine J. Vinknes

**Affiliations:** 1 Department of Nutrition, Institute of Basic Medical Sciences, University of Oslo, 0372 Oslo, Norway; benteov@me.com (B.Ø.); n.e.bastani@medisin.uio.no (N.E.B.); helga.refsum@medisin.uio.no (H.R.); kathrine.vinknes@medisin.uio.no (K.J.V.); 2 Øvrebø Nutrition, 0550 Oslo, Norway; 3 Department of Pharmacology, University of Oxford, Oxford OX1 3QT, UK; cheryl.turner@pharm.ox.ac.uk

**Keywords:** methionine, cysteine, amino acids, steaoryl-CoA desaturase, clinical trial

## Abstract

Dietary and plasma total cysteine (tCys) have been associated with adiposity, possibly through interaction with stearoyl–CoA desaturase (SCD), which is an enzyme that is involved in fatty acid and energy metabolism. We evaluated the effect of a dietary intervention with low cysteine and methionine and high polyunsaturated fatty acids (PUFAs) on plasma and urinary sulfur amino acids and SCD activity indices. Fourteen normal-weight healthy subjects were randomized to a seven-day diet low in cysteine and methionine and high in PUFAs (Cys/Met_low_ + PUFA), or high in saturated fatty acids (SFA), cysteine, and methionine (Cys/Met_high_ + SFA). Compared with the Cys/Met_high_ + SFA group, plasma methionine and cystathionine decreased (*p*-values < 0.05), whereas cystine tended to increase (*p* = 0.06) in the Cys/Met_low_ + PUFA group. Plasma total cysteine (tCys) was not significantly different between the groups. Urinary cysteine and taurine decreased in the Cys/Met_low_ + PUFA group compared with the Cys/Met_high_ + SFA group (*p*-values < 0.05). Plasma SCD-activity indices were not different between the groups, but the change in cystine correlated with the SCD-16 index in the Cys/Met_low_ + PUFA group. A diet low in methionine and cysteine decreased plasma methionine and urinary cysteine and taurine. Plasma tCys was unchanged, suggesting that compensatory mechanisms are activated during methionine and cysteine restriction to maintain plasma tCys.

## 1. Introduction

There is increasing evidence on the importance of dietary protein in the regulation of metabolism and body adiposity, and a high habitual intake of predominantly animal protein is associated with increased adiposity in both cross-sectional and prospective studies [1,2,3,4,5,6,7]. This is consistent with human studies observing that plant-based diets are associated with the prevention of weight gain, type 2 diabetes, and cardiovascular disease [6,8,9].

Methionine and cysteine are sulfur amino acids (SAAs) that are found in low amounts in plant foods and higher amounts in meats and fish [10]. Methionine is an essential amino acid that must be obtained from the diet, whereas cysteine is classified as conditionally essential, and can be obtained from the diet or synthesized endogenously from methionine metabolism [11,12]. More specifically, SAA metabolism includes the conversion of methionine to homocysteine through a sequence of reactions that are commonly termed transmethylation. Homocysteine can then undergo irreversible transsulfuration in the liver to form cystathionine and ultimately cysteine [11], which may be utilized in the synthesis of protein, taurine, or the antioxidant glutathione. Furthermore, cysteine is readily oxidized to form a dimer containing a disulfide bond between two cysteine molecules, cystine, or with other thiols (including thiols in protein), thereby forming cysteine mixed disulfides.

Total cysteine (tCys) refers to the sum of cysteine equivalents available after reduction of its oxidized forms. In plasma, it mainly consists of protein-bound cysteine, free cysteine-mixed disulfides, cysteine, and reduced cysteine (thiol form). Plasma concentrations of tCys have been strongly and positively associated with fat mass and obesity in several large epidemiological studies [13,14,15]. Moreover, results from studies examining the association of plasma SAAs with body mass index (BMI) and fat mass in children and adults [14,16,17] suggest that these associations are mainly confined to tCys [12,18].

The diet or drug-induced lowering of plasma tCys concentrations has been associated with beneficial changes in body composition in animal models [19,20,21,22], but the mechanisms through which cysteine promotes obesity is presently unknown. One possible mechanism may be by influencing the fatty acid desaturase enzyme stearoyl–CoA desaturase-1 (SCD1). In rodents, diets with low methionine/cysteine or excess cysteine respectively suppress or enhance hepatic *Scd1* expression, plasma protein levels, and plasma SCD activity indices (fatty acid ratios of C16:1n-7/C16:0 and C18:1n-9/C18:0) [19,23]. Additionally, plasma tCys was positively associated with plasma SCD activity indices in two human populations [24]. Plasma SCD indices have previously been associated with obesity [25,26], and *Scd1* expression is down-regulated by the intake of polyunsaturated fatty acids (PUFA) in mice [27,28].

In this pilot study, we aimed to test a diet specifically developed for influencing SCD activity indices. We designed a diet low in methionine and cysteine and high in PUFAs and one diet high in methionine, cysteine and saturated fatty acids (SFA), and thus attempted to maximally influence SCD activity indices in plasma. The contents of cysteine and methionine in the two dietary interventions were set to reflect a diet profoundly restricted in SAAs (plant-based diet) versus a diet higher in protein (animal-based diet) [10]. We then compared the effect of a seven-day dietary intervention with contrasting contents of methionine and cysteine and PUFA on SAA concentrations in plasma and urine. Moreover, we assessed whether the diets influenced SCD activity indices in the plasma of healthy individuals, and whether changes in plasma SAAs were associated with the changes in SCD plasma activity indices.

## 2. Materials and Methods

### 2.1. Participants

In total, 14 participants were recruited online through the web and social media page of the University of Oslo. Since this was a pilot study, sample size was not calculated prior to trial commencement. Inclusion criteria were healthy normal-weight (BMI 20–25 kg/m^2^) men and women, aged 20–40 years. Exclusion criteria were high intake of fatty fish or cod liver oil (fatty fish ≥four times per week, cod liver oil, or n-3 fatty acid supplements ≥three times every week), the use of medications, smoking (daily), the presence of major chronic disease, regular alcohol consumption (defined as ≥four times per week), pregnancy, and breastfeeding. Written informed consent was obtained from all of the participants. The study protocol was approved by the Regional Committee for Medical Research Ethics South East and was registered with the United States (US) National Library of Medicine Clinical Trials registry (ClinicalTrials.gov Identifier: NCT02647970, registration date: 6 January 2016). All aspects of the study were conducted according to the Declaration of Helsinki.

### 2.2. Study Design and Outcomes

The experimental protocol followed a randomized, parallel-design intervention trial among healthy volunteers. The trial consisted of a full diet intervention for seven days with follow-up visits at day three and day seven. The study was conducted at Centre for Clinical Nutrition at Institute of Basic Medical Sciences, University of Oslo (Oslo, Norway).

To ensure the similar distribution of men in each group, stratified block randomization with a block size of four was carried out using the “blockrand” package for R 3.0.2 (the R Foundation for Statistical Computing, Vienna, Austria) by study personnel. In total, 14 participants were randomized to one of two interventions either 1) rich in PUFA and low in cysteine and methionine (Cys/Met_low_ + PUFA) (*n* = 7) or 2) rich in SFA, cysteine, and methionine (Cys/Met_high_ + SFA) (*n* = 7). Primary outcomes were plasma SAAs and SCD activity, which was measured as plasma fatty acid product/precursor ratios. 

One week before trial commencement, the participants were instructed to completely avoid intake of fatty fish, cod liver oil, n-3 fatty acid supplements, and other supplements, and consume only moderate amounts of alcohol (maximum two units three times per week). They were informed to keep their physical activity level normal, but avoid strenuous physical activity the day before the start of the study and during the intervention period. In addition, the subjects had to abstain from alcohol 24 h before the start of the study, and were instructed to eat a light meal low in fat after 18 h, and not to eat anything eight hours before blood sampling at baseline.

### 2.3. Dietary Interventions

We developed a diet high in PUFA (n-3 and n-6 fatty acids) and low in SAAs and a control diet containing SFA and SAAs similar to a typical western diet. Cysteine and methionine are abundant in animal-derived protein, but also exist in certain fruits, grains, and vegetables; thus, both diets were vegan-based without meat, fish, eggs, dairy products, and certain plant-based foods, but included butter as the primary fat source in the Cys/Met_high_ + SFA diet. In order to achieve the recommended protein and micronutrient intake [29], all of the participants were given a powdered drink mix without SAAs (XMET XCYS Maxamaid^®^ provided by Nutricia Norway AS, Oslo, Norway) that contained other essential and non-essential amino acids, carbohydrate, vitamins, minerals, and trace elements. Powdered SAAs (Jo Mar Laboratories, Scotts Valley, CA, USA) were added to the Cys/Met_high_ + SFA drink mix. Both diets also included n-3 supplements (Triomar^®^ (Orkla Health Norge, Oslo, Norway) containing 1.32 g of n-3 and Møllers Dobbel Kapsel^®^ (Orkla Health Norge, Oslo, Norway) containing 0.4 g of n-3, vitamin A (139 µg), vitamin D (8.3 µg), and vitamin E (5.6 mg)). Meals were adapted to a Norwegian cuisine and available ingredients. 

The supplements and foods were delivered to the home address of each participant, including a menu with recipes for each meal and information on daily intake. In an attempt to blind the participants to the intervention, the only difference between the two diets was the fat source used and the addition of SAAs to the powdered drink mix in the Cys/Met_high_ + SFA diet. A typical daily menu is outlined in Supplementary Table S1, and consisted of three main meals (breakfast, lunch, and dinner), one snack, and four glasses of juice mixed with the powdered drink mix. Both diets consisted of 100 g/d of the powdered drink mix without SAAs. In the Cys/Met_high_ + SFA diet, a total of 4.8 g of SAAs (3.2 g cystine, 1.6 g methionine) were added to this amount. The participants were instructed to mix the powder with grape juice and drink it with each meal (4/d, ~25 g/meal). The energy content of the diets was adapted to average gender requirements [29], and corresponded to 2000 kcal/d for women and 2500 kcal/d for men. The relative contribution of fat to the Cys/Met_low_ + PUFA diet was 5.5 energy % (E%) SFA and 10.9 E% PUFA (5.16 g n-3 and 18.3 g n-6) for women and 5.2 E% SFA and 10.6 E% PUFA (5.86 g n-3 and 22.1 g n-6) for men. The total SAA content was 0.93 g for women and 1.19 g for men, which was considered restricted compared to a diet with higher protein content [10]. In the Cys/Met_high_ + SFA group, the relative contribution was 13.5 E% SFA and 3.3 E% PUFA (1.5 g n-3 and 4.97 g n-6) for women, and 13.3 E% SFA and 3.21 E% PUFA (1.8 g n-3 and 6.17 g n-6) for men. The total SAA content was 5.75 g for women and 6.01 g for men, which is in line with a diet with higher protein content [10]. A detailed overview of the nutrient composition, including the SAA and fatty acid contents of the seven-day diet is presented in Supplementary Table S2. Participants were permitted to drink unlimited amounts of coffee, tea, water, and diet soda. 

### 2.4. Data Collection

#### 2.4.1. Lifestyle and Dietary Data

Data on health status and disease, the use of drugs, vitamins, or supplements, dietary habits, physical activity, cigarette smoking, alcohol consumption, and education were recorded using self-administered online questionnaires.

#### 2.4.2. Anthropometric Parameters

A bioelectrical impedance weight (Seca mBCA515, Hamburg, Germany) was used to measure body weight and calculate BMI.

### 2.5. Blood and Urine Sampling and Biochemical Assays

#### 2.5.1. Blood Sampling

Venous blood samples were collected from each participant at days 0, three, and seven after an overnight fast. Blood from each participant was collected into three ethylenediaminetetetraacetic acid (EDTA)-lined vacuum tubes including one tube containing N-ethylmalemide (NEM) 150 mmol/L at 10% of the volume of the tube. NEM was used as a derivatization reagent to immediately trap the thiols [30]. Immediately after withdrawal, the blood was centrifuged for five minutes at 4 °C. Plasma from the EDTA tubes without NEM were handled in two ways. (1) Aliquots of plasma were stored immediately at −80 °C until analysis for total amino acids and fatty acids, and (2) aliquots were precipitated with 5-sulfosalicyclic acid (SSA) 10% to a final concentration of 5%. The resultant supernatant was aliquoted and stored at −80 °C until analysis of S-adenosylmethionine (SAM), S-adenosylhomocysteine (SAH), free homocysteine, cysteine, and glutathione. Plasma aliquots from the tubes containing both EDTA and NEM were also precipitated with SSA 10%. The resultant supernatant was aliquoted and stored at −80 °C until analysis of free oxidized and reduced homocysteine, cysteine, and glutathione. 

#### 2.5.2. Urine Sampling

Fasting morning urine samples were obtained at baseline, days three and seven. Urine samples were delivered in 100-mL cups and subsequently distributed in 9.5-mL tubes and frozen at −80 °C until analysis.

#### 2.5.3. Clinical Biochemistry

Blood samples for the measurement of routine clinical biochemistry parameters were collected in EDTA-lined vacuum tubes and in gel tubes containing lithium heparin. Plasma concentrations of total cholesterol, low-density lipoprotein (LDL) cholesterol (LDL-C), high-density lipoprotein (HDL)-C, and triglycerides were measured at the Department of Medical Biochemistry (Oslo University Hospital Rikshospitalet, Oslo, Norway) by colorimetric and/or enzymatic methods on a Cobas c702 analyzer (Roche Diagnostics International Ltd., Rotkreuz, Switzerland).

#### 2.5.4. Amino Acid Assays

Total (plasma and urine), free reduced/oxidized (plasma), and unbound (plasma) methionine, homocysteine, cysteine, cystathionine, taurine, and glutathione, as well as SAM and SAH, were measured by liquid chromatography–tandem mass spectrometry (LC-MS/MS). LC-MS/MS was performed using a Shimadzu LC-20ADXR Prominence LC system (Kyoto, Japan) coupled to a Sciex QTRAP5500 mass spectrometer with a Turbo V ion source and TurboIonspray probe (Framingham, MA, USA). The separation of the analytes was achieved using a Phenomenex Kinetex 2.6 μm C18 100A 100 × 4.6 mm column with an aqueous solution of formic acid (0.5%) and heptafluorobutyric acid (0.3%) and acetonitrile gradient mobile phase. Linear calibration curves of the peak area ratios of analytes and internal standards were used for quantification. Modified versions of previously described methods were used for total [31] and free reduced/oxidized forms [32] of amino acids as discussed briefly below. 

For total plasma and urinary SAAs, isotopically labeled internal standards were added to plasma or urine followed by the reduction of disulfides using dithioerythritol (DTE) 100 mmol/L, and then protein precipitation with SSA 10%. The extracts were diluted with an aqueous solution of formic acid [0.5%] and heptafluorobutyric acid [0.3%] prior to analysis. Coefficients of variation for the analytes were 3.4–6.7%. For plasma free reduced and oxidized cysteine and homocysteine, the NEM containing acidic plasma extracts were used. Cysteine-NEM, homocysteine-NEM, cysteine, and homocysteine were measured with isotopically labeled internal standards. Plasma SAM and SAH were measured from the acidic plasma extracts using isotopically labeled internal standards. The coefficients of variation for the analytes were 2–3%. The acidic plasma extracts were also used to measure the unbound fraction. The acidic extracts were neutralized with ammonia [0.25 mol/L] prior to the addition of the reductant DTE. The total unbound fraction represents the combined free reduced and disulfide concentrations (including mixed disulfides). The coefficients of variation of the analytes were 4–6%. The bound fraction was calculated by subtracting the unbound concentration from the total plasma concentration.

#### 2.5.5. Fatty Acid Assays

Total plasma fatty acid concentrations were measured by gas chromatography—mass spectrometry (GC-MS) using a modified version of a previously described method [33]. The method involves the in situ transesterification of total lipid fatty acids from plasma into methyl esters. The fatty acid methyl esters (FAME) were analyzed by GC-MS. Briefly, methanolic hydrochloric acid (3*N*) was added to plasma and incubated in a water bath at 85 °C for 1.5 h. Heneicosanoic acid (C21:0) was used as an internal standard. Hexane containing butylated hydroxytoluene as an antioxidant was used for extraction. The hexane fraction containing FAME was analyzed using a Focus GC-DSQ II GC-MS system (Waltham, MA, USA) with an SGE GC column BPX70 25 m × 0.22 mm, with 0.25-μm film (Victoria, Australia). Electron ionization positive mode selective ion monitoring was used for detection. Linear calibration curves of the peak area ratios of the analyte and internal standard were used for quantification. The coefficient of variation was 4.5–11.5%. SCD indices were given as product/precursor-ratios of fatty acids in plasma (SCD-16; C16:1n-7/C16:0, SCD-18; C18:1n-9/C18:0).

### 2.6. Statistical Analysis

Data were presented as medians (range) for continuous variables and counts (%) for categorical variables unless otherwise specified. Due to the small sample size and non-normal data distribution, we used non-parametric statistical tests for our main analyses. The Wilcoxon rank-sum test was used to assess between-group differences for continuous variables at baseline, and additionally at day three and day seven. Interventions were compared using linear mixed model regression (LMM). The models included group, time, and an interaction term for group and time as fixed effects. The interaction term was included in order to test for differences between groups depending on the time. Subjects were included as random effect variables to adjust for within-subject correlation. Due to the low number of participants, baseline differences occurred for plasma total homocysteine, reduced homocysteine, and homocysteine. These analyses were adjusted accordingly by including only group and the interaction term for group and time in the model for these parameters. The LMM approach provides several advantages over traditional repeated measures analysis of variance, as demonstrated elsewhere [34]. Results are expressed as estimated marginal means (EMMs) at each time point, and their corresponding 95% confidence intervals were derived from each model. Simple linear regression was conducted to examine the relationship of SAAs with fatty acids and SCD activity indices. These analyses were conducted separately for each group or for the groups combined, adjusting for intervention allocation. The significance level was set to *p* < 0.05. Statistical analyses were carried out using the “base” and “nlme” packages for the R (the R Foundation for Statistical Computing, Vienna, Austria) interface in the LabKey Server (Nelson EK, BMC Bioinformatics, 2011). 

## 3. Results

### 3.1. Baseline Characteristics

**Figure 1:**
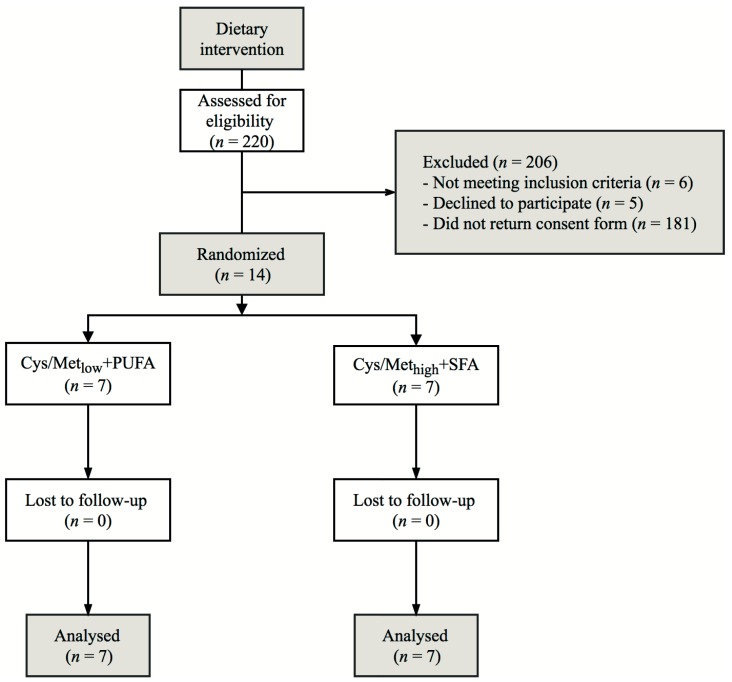
Flowchart outlining the study design and progression. Cys/Met_low_+PUFA, diet low in cysteine and methionine and high in polyunsaturated fatty acids; Cys/Met_high_+SFA, diet high in cysteine, methionine and saturated fatty acids.

Recruitment and completion of the trial was performed from May to November 2016. A flowchart outlining the study design and the flow of participants is presented in Figure 1. Baseline characteristics of the participants are presented in Table 1. Each group consisted of five women and two men. All of the randomized participants completed the trial. The groups were similar at baseline except for plasma total cholesterol, which was higher in the Cys/Met_low_ + PUFA group compared with the Cys/Met_high_ + SFA group. For subsequent analyses, all of the data from all 14 participants were available. Changes in baseline characteristics from day 0 to 7 are presented in Supplementary Table S3. Except for a trend for a greater reduction in total cholesterol in the Cys/Met_low_ + PUFA group compared with the Cys/Met_high_ + SFA group (*p* = 0.055), no significant differences in change for the baseline characteristics were observed.

### 3.2. Changes in Plasma SAAs in Response to the Dietary Interventions

EMMs, their corresponding confidence intervals, and *p*-values for plasma concentrations of the SAAs at baseline, day three, and day seven are presented in Table 2 and Table 3. We observed significant group×time interactions for methionine, SAH, total homocysteine, and cystathionine. Overall, methionine and cystathionine decreased, whereas SAH and total homocysteine increased from baseline to day seven in the Cys/Met_low_ + PUFA group compared to the Cys/Met_high_ + SFA group. We observed a trend for a group×time interaction for cysteine, which increased in the Cys/Met_low_ + PUFA group compared to the Cys/Met_high_ + SFA group (*p* = 0.06).

### 3.3. Changes in Urine SAAs in Response to the Dietary Interventions

EMMs, their corresponding confidence intervals, and *p*-values for urinary SAAs at each visit are presented in Table 4. We observed a significant group×time interaction for methionine, cysteine, and taurine, all of which were excreted to a lower extent in the Cys/Met_low_ + PUFA group compared to the Cys/Met_high_ + SFA group after seven days.

### 3.4. SCD Indices Based on Plasma Total Fatty Acids

EMMs, their corresponding confidence intervals a *p*-values for C16:1n-7, C16:0, C18:1n-9, and C18:0, as well as SCD-16 and SCD-18, are presented in Supplementary Table S3. No significant interactions were observed following the dietary intervention for the fatty acids or SCD plasma indices.

### 3.5. Associations between tCys Fractions and SCD Indices

To further examine the relationship between SAAs and fatty acids, we performed simple linear regression to assess whether the change in tCys and its fractions were associated with changes in SCD activity indices. In a model adjusted for intervention allocation, change in cystine (standardized β = 0.72, 95% CI: 0.25–1.20, *p* = 0.014) and reduced cysteine (standardized β = 0.59, 95% CI: 0.10–1.10, *p* = 0.04) was positively associated with the SCD-16 index. Similar results were observed only in the Cys/Met_low_ + PUFA group (data not shown).

### 3.6. Harmful Effects

No harmful effects of the dietary interventions were reported. Two participants, one for each group, reported light gastrointestinal problems.

## 4. Discussion

In this randomized clinical trial and pilot study, we demonstrated effects of a seven-day diet rich in PUFA and restricted in methionine and cysteine on plasma and urinary concentrations of SAA and related products from SAA metabolism.

To our knowledge, this is the first study to address the effects of combined SAA restriction and PUFA enrichment on plasma concentrations of SAAs and fatty acids. We observed no significant differences in plasma tCys between the Cys/Met_low_ + PUFA and Cys/Met_high_ + SFA group. However, there was a somewhat surprising but clear trend for an increase in plasma cystine and reduced cysteine, which are important fractions of tCys [30], in the Cys/Met_low_ + PUFA compared to the Cys/Met_high_ + SFA group. Although there are other studies on diets where SAA intake was manipulated [35,36,37], our results are not directly comparable due to methodological differences such as differing contents of protein and SAA in the diets. However, one study that examined the effects of dietary SAA depletion on fasting plasma cysteine concentrations showed decreased plasma free cysteine concentrations, but no change in free cystine after four days of SAA depletion [38]. The absent effect of SAA restriction on plasma tCys in our study, and the trend for increased cystine concentrations, suggest that compensatory mechanisms are activated when dietary methionine and cysteine are limited. In particular, the enzyme cysteine dioxygenase, which catalyzes the irreversible two-step conversion of cysteine to taurine, possesses negligible activity in rats fed an SAA-deficient diet, and is considered essential for the maintenance of plasma tCys and sulfur availability [39,40]. We also demonstrated a significant decline in the urinary excretion of cysteine in the Cys/Met_low_ + PUFA group compared to the Cys/Met_high_ + SFA group in which excretion increased considerably. This is in line with a previous study that demonstrated decreased urinary sulfate excretion following protein restriction when comparing diabetics to healthy controls [41]. Thus, we speculate that compensatory mechanisms resulted in increased plasma cystine concentrations and a lower urinary output of tCys, indicating that urinary tCys excretion may be a more suitable marker for dietary cysteine restriction compared to plasma tCys. However, the relevance of this particular finding is unclear, and should be addressed further as it is not known whether urinary tCys resembles plasma tCys in its association with adverse outcomes.

We observed an increase in plasma homocysteine in the Cys/Met_low_ + PUFA group compared to the Cys/Met_high_ + SFA group. The increase in homocysteine could be explained by the allosteric regulation of cystathionine-β-synthase by SAM, resulting in the inhibition of transsulfuration and thereby increased homocysteine concentrations available for homocysteine remethylation to methionine. Although circulating homocysteine has been recognized as a risk factor for cardiovascular disease [42], the increase in the Cys/Met_low_ + PUFA group possibly reflects a homeostatic response to maintain plasma methionine in order to conserve methionine for protein synthesis and SAM production [43]. SAM is the primary methyl group donor in the majority of biological methylation reactions, including DNA methylation. The product of these methylation reactions is SAH, which is a potent inhibitor of transmethylations, and the ratio of SAM/SAH is regarded as the measure of methylation capacity [44]. Interestingly, SAH was increased in the Cys/Met_low_ + PUFA group compared to the Cys/Met_high_ + SFA group. Both deficient and excess methionine intake can impact the methionine cycle, resulting in alterations of SAM, SAH, and homocysteine concentrations, with a possible impact on DNA methylation and consequently gene expression.

The dietary intervention did not affect plasma indices of SCD-16 or SCD-18. However, we observed a positive association between the change in cystine and reduced cysteine and the change in the SCD-16 index from baseline to day seven in the Cys/Met_low_ + PUFA group. Animal studies have shown that cysteine affects SCD expression in the liver [19,23], and a positive association between tCys and SCD has been demonstrated in human populations [24]. This finding indicates that cysteine restriction induces cysteine-sparing effects, which in turn may affect plasma SCD activity indices. Future studies should address whether this effect is present in interventions with SAA restriction beyond one week. 

The strengths of this study include the randomized, controlled design of the intervention and a well-balanced diet independent of the interventions. Moreover, although we did not manage to fully blind the different tastes of the interventions, the only differing foodstuffs between the diets was the oil/butter used as fat sources in some of the meals, and the powdered methionine and cysteine supplied in the drink mix of the participants in the SFA + Cys/Met group. The level of cysteine and methionine in the Cys/Met_low_ + PUFA group corresponded to ~19% of the level in the Cys/Met_high_ + SFA group. In comparison, a common methionine-restricted diet in rodents is deficient in cysteine and contains a methionine level of ~20% relative to the control group [45]. This study also has several limitations, including a small sample size and short observation period, and it is possible that the null effect on plasma concentrations of tCys and SCD, as well as the increase in cysteine was influenced by the short intervention period. One 16-week study in overweight individuals managed to lower cysteine [46], and the trend for an increase that we observed may be transient and reflect short-term compensatory mechanisms. Considering that SCD is normally responsive to PUFA intake in humans [47,48,49], we cannot exclude the possibility that the recruited individuals were already eating high amounts of PUFAs. Although we excluded participants with very high intakes of PUFA-rich foods or supplements, the intervention may thus not have been intensive enough to induce a response in plasma. The diet was created to maximize the effects on SCD activity indices; however, we acknowledge that a factorial 2 × 2 design may be preferable to the current design, as the effects of methionine and cysteine restriction cannot be distinguished from the increased PUFA content. Thus, future investigations should be of longer term and with careful selection of an appropriate study population, and the intervention design should be reconsidered. Finally, there were relatively large baseline differences in some of the metabolites, including homocysteine, which may be attributed to unfortunate randomization. We attempted to adjust for baseline differences in the analyses, but cannot exclude the possibility of additional confounding introduced by errors in the randomization protocol. 

We show that plasma methionine and cystathionine, and urinary cysteine and taurine were decreased, whereas plasma cysteine and markers of SCD activity were not affected following a seven-day diet low in methionine and cysteine. Future studies should assess the potential compensatory mechanisms in response to SAA restriction, and whether these findings have implications for clinical end points. Furthermore, urinary levels of cysteine and taurine should be further investigated as potential biomarkers for dietary SAA intake. In order to establish the clinical implications of a diet low in SAA and high in PUFA, future studies should assess its effects in an overweight or obese population in which SAA and SCD1 activity indices are likely to be higher, possibly reflecting a disrupted metabolism of these compounds.

## Figures and Tables

**Table 1:** Characteristics of the study population

	Cys/Met_low_ + PUFA	Cys/Met_high_ + SFA	*p*
Female, n	5	5	-
Male, n	2	2	-
Age, y	31 (20–37)	24 (21–38)	0.70
Weight, kg	66.8 (59.2–83.9)	65.7 (59.5–80.2)	0.90
Height, m	1.71 (1.66–1.89)	1.73 (1.59–1.84)	0.75
BMI, kg/m^2^	22.6 (21.1–25.0)	22.3 (20.7–26.2)	1.00
Total cholesterol, mmol/L	4.4 (4.0–4.8)	3.8 (2.9–5.4)	0.04
HDL cholesterol, mmol/L	1.6 (1.3–2.1)	1.5 (1.2–2.3)	0.84
LDL cholesterol, mmol/L	2.5 (2.0–2.6)	2.0 (1.4–2.9)	0.60
Triglycerides, mmol/L	0.7 (0.5–1.0)	0.8 (0.4–1.0)	1.00

Data are expressed as medians (range) or numbers. The Wilcoxon rank-sum test was used to assess between-group differences for continuous variables at baseline. Cys/Met_low_+PUFA, diet low in cysteine and methionine and high in polyunsaturated fatty acids; Cys/Met_high_+SFA, diet high in cysteine, methionine and saturated fatty acids; BMI, body mass index, HDL, high-density lipoprotein; LDL, low-density lipoprotein.

**Table 2:** Estimated marginal means (95% confidence interval) of plasma concentrations of amino acids and related metabolites.

Amino Acids	Group	Baseline	Day 3	Day 7	*p*_int_
Methionine ^1^	Cys/Met_low_ + PUFA	23.6 (21.2, 26)	22.4 (20.1, 24.8)	21.1 (18.7, 23.5)	0.044
	Cys/Met_high_ + SFA	22.2 (19.8, 24.6)	21.8 (19.5, 24.2)	23 (20.6, 25.3)	
SAM ^1^	Cys/Met_low_ + PUFA	102 (92.9, 110)	110 (101, 119)	110 (101, 119)	0.27
	Cys/Met_high_ + SFA	104 (95.3, 113)	100 (91.8, 109)	105 (95.9, 113)	
SAH ^1^	Cys/Met_low_ + PUFA	12.8 (8.8, 16.8)	14.2 (10.2, 18.2)	14 (10, 18)	< 0.01
	Cys/Met_high_ + SFA	15.3 (11.3, 19.3)	15.2 (11.2, 19.2)	13.1 (9.14, 17.1)	
SAM/SAH	Cys/Met_low_ + PUFA	8.06 (6.56, 9.57)	7.94 (6.44, 9.45)	7.98 (6.47, 9.49)	0.25
	Cys/Met_high_ + SFA	7.8 (6.29, 9.3)	7.6 (6.1, 9.11)	8.42 (6.91, 9.92)	
tHcy ^1^	Cys/Met_low_ + PUFA	10.1 (8.77, 11.5)	11.6 (10.3, 13)	11.2 (9.83, 12.5)	< 0.01
	Cys/Met_high_ + SFA	6.79 (5.44, 8.14)	6.44 (5.09, 7.79)	6.28 (4.93, 7.63)	
Cystathionine ^2^	Cys/Met_low_ + PUFA	249 (186, 311)	104 (40.8, 166)	78.3 (15.5, 141)	0.041
	Cys/Met_high_ + SFA	110 (46.7, 172)	117 (53.8, 179)	126 (63.4, 189)	
tCys ^1^	Cys/Met_low_ + PUFA	249 (221, 276)	268 (240, 295)	262 (234, 289)	0.35
	Cys/Met_high_ + SFA	241 (214, 269)	251 (223, 278)	246 (218, 273)	
GSH ^1^	Cys/Met_low_ + PUFA	5.5 (4.75, 6.25)	6.29 (5.54, 7.04)	4.91 (4.16, 5.66)	0.51
	Cys/Met_high_ + SFA	5.62 (4.87, 6.37)	5.96 (5.21, 6.71)	4.63 (3.88, 5.38)	
Taurine ^1^	Cys/Met_low_ + PUFA	87.2 (69.2, 105)	107 (89.1, 125)	74.5 (56.4, 92.5)	0.46
	Cys/Met_high_ + SFA	77.5 (59.5, 95.6)	82.4 (64.4, 100)	75.7 (57.6, 93.7)	

Estimated marginal means and their 95% confidence intervals of amino acids at baseline, day three, and day seven in diet interventions. *p*-values are computed using linear mixed model regression with group, time, and group×time interaction term in the model. Cys/Met_low_+PUFA, diet low in cysteine and methionine and high in polyunsaturated fatty acids; Cys/Met_high_+SFA, diet high in cysteine, methionine and saturated fatty acids; *p*_int_, *p*-value for group×time interaction. Abbreviations: SAM, S-adenosylmethionine; SAH, S-adenosylhomocysteine; tHcy, total homocysteine; tCys, total cysteine; GSH, total glutathione. ^1^ Values expressed as μmol/L; ^2^ values expressed as nmol/L.

**Table 3:** Estimated marginal means (95% confidence interval) of fractions of total homocysteine and total cysteine in plasma.

Amino Acids	Group	Baseline	Day 3	Day 7	*p*_int_
Free hcy ^1^	Cys/Met_low_ + PUFA	2.77 (2.47, 3.08)	3.00 (2.70, 3.31)	2.95 (2.64, 3.26)	0.023
	Cys/Met_high_ + SFA	1.96 (1.66, 2.27)	1.83 (1.52, 2.14)	1.89 (1.58, 2.2)	
Protein-bound hcy ^1^	Cys/Met_low_ + PUFA	7.34 (6.25, 8.43)	8.61 (7.53, 9.7)	8.23 (7.14, 9.31)	< 0.01
	Cys/Met_high_ + SFA	4.82 (3.74, 5.91)	4.61 (3.52, 5.7)	4.39 (3.3, 5.48)	
Red hcy ^2^	Cys/Met_low_ + PUFA	141 (93, 189)	156 (108, 204)	184 (134, 234)	0.29
	Cys/Met_high_ + SFA	172 (124, 220)	136 (88.3, 184)	174 (126, 222)	
Homocystine ^2^	Cys/Met_low_ + PUFA	22.9 (17.7, 28.2)	30.3 (25.1, 35.6)	29.6 (24.1, 35.1)	< 0.01
	Cys/Met_high_ + SFA	12.8 (7.49, 18)	11.4 (6.09, 16.6)	11.3 (6.08, 16.6)	
Reduced hcy/homocystine	Cys/Met_low_ + PUFA	6.71 (3.48, 9.95)	5.5 (2.27, 8.74)	6.5 (3.12, 9.88)	0.48
	Cys/Met_high_ + SFA	14.1 (10.9, 17.4)	11.8 (8.57, 15)	15.6 (12.4, 18.8)	
Free cysteine ^1^	Cys/Met_low_ + PUFA	118 (106, 131)	121 (108, 134)	122 (109, 134)	0.55
	Cys/Met_high_ + SFA	115 (102, 128)	116 (103, 129)	121 (108, 134)	
Protein-bound cysteine ^1^	Cys/Met_low_ + PUFA	130 (114, 147)	147 (130, 163)	140 (124, 157)	0.21
	Cys/Met_high_ + SFA	126 (110, 143)	135 (118, 151)	125 (108, 141)	
Reduced cysteine ^1^	Cys/Met_low_ + PUFA	10.1 (8.44, 11.8)	9.65 (7.96, 11.3)	10.2 (8.46, 12)	0.42
	Cys/Met_high_ + SFA	12.5 (10.8, 14.2)	11.2 (9.55, 12.9)	13.7 (12, 15.4)	
Cystine ^1^	Cys/Met_low_ + PUFA	35.5 (30.8, 40.1)	37.4 (32.7, 42)	39 (34.2, 43.8)	0.06
	Cys/Met_high_ + SFA	36.1 (31.5, 40.8)	36.7 (32.1, 41.4)	35.5 (30.8, 40.2)	
Reduced cysteine/cystine	Cys/Met_low_ + PUFA	0.291 (0.24, 0.34)	0.262 (0.21, 0.31)	0.265 (0.21, 0.32)	0.07
	Cys/Met_high_ + SFA	0.348 (0.30, 0.40)	0.31 (0.26, 0.36)	0.389 (0.34, 0.44)	

Estimated marginal means and their 95% confidence intervals of homocysteine, cysteine and glutathione at baseline, day three, and day. *p*-values are computed using linear mixed model regression with group, time, and group×time interaction term in the model. Cys/Met_low_+PUFA, diet low in cysteine and methionine and high in polyunsaturated fatty acids; Cys/Met_high_+SFA, diet high in cysteine, methionine and saturated fatty acids; *p*_int_, *p*-value for group×time interaction. Hcy, homocysteine; ^1^ Values expressed as μmol/L; ^2^ values expressed as nmol/L.

**Table 4:** Estimated marginal means (95% confidence interval) of urinary concentrations of amino acids and related metabolites.

Amino Acids, μmol/mmol Creatinine		Baseline	Day 3	Day 7	*p*_int_
Methionine	Cys/Met_low_ + PUFA	0.83 (0.63, 1.04)	0.44 (0.20, 0.67)	0.38 (0.18, 0.58)	0.047
	Cys/Met_high_ + SFA	0.80 (0.60, 1.00)	0.65 (0.45, 0.85)	0.73 (0.53, 0.93)	
tHcy	Cys/Met_low_ + PUFA	0.31 (0.17, 0.45)	0.34 (0.18, 0.52)	0.29 (0.16, 0.43)	0.316
	Cys/Met_high_ + SFA	0.22 (0.09, 0.36)	0.32 (0.19, 0.46)	0.33 (0.19, 0.46)	
Cystathionine	Cys/Met_low_ + PUFA	4.27 (2.72, 5.81)	0.18 (−1.65, 2.01)	0.182 (−1.37, 1.73)	0.043
	Cys/Met_high_ + SFA	0.86 (−0.68, 2.42)	0.68 (−0.86, 2.24)	0.93 (−0.61, 2.48)	
tCys	Cys/Met_low_ + PUFA	21.2 (16.2, 26.2)	18.1 (12.5, 23.6)	17.9 (12.9, 22.9)	0.001
	Cys/Met_high_ + SFA	21.1 (16.1, 26.1)	30.4 (25.4, 35.4)	32.2 (27.2, 37.2)	
Glutathione	Cys/Met_low_ + PUFA	0.12 (0.06, 0.18)	0.10 (0.03, 0.17)	0.09 (0.03, 0.15)	0.786
	Cys/Met_high_ + SFA	0.21 (0.15, 0.27)	0.21 (0.15, 0.27)	0.17 (0.11, 0.22)	
Taurine	Cys/Met_low_ + PUFA	42.6 (23.8, 61.3)	23.8 (2.28, 45.4)	8.72 (−10.0, 27.5)	0.009
	Cys/Met_high_ + SFA	15.9 (−2.81, 34.7)	19.9 (1.12, 38.6)	23.6 (4.82, 42.3)	

Estimated marginal means and their 95% confidence intervals of creatinine adjusted urinary concentrations amino acids at baseline, day three, and day seven in diet interventions with low methionine and cysteine and high PUFA, or high methionine, cysteine, and SFA. *p*-values are computed using linear mixed model regression with group, time, and group×time interaction terms in the model. Cys/Met_low_+PUFA, diet low in cysteine and methionine and high in polyunsaturated fatty acids; Cys/Met_high_+SFA, diet high in cysteine, methionine and saturated fatty acids; *p*_int_, *p*-value for group×time interaction. tHcy, total homocysteine; tCys, total cysteine.

## Data Availability

Raw data material and protocol will be made available upon request to the authors.

## Supplementary Materials

The following are available online at http://www.mdpi.com/2072-6643/10/12/1822/s1, Table S1: A typical daily menu in the 7-day diet; Table S2: Average nutrient content and composition in the 7-day diet for each intervention group; Table S3: Change from baseline in metabolic parameters; Table S4: Fatty acids and SCD activity indices.
