# Supplementary material for: Combining Dietary Sulfur Amino Acid Restriction with Polyunsaturated Fatty Acid Intake in Humans: A Randomized Controlled Pilot Trial
Source: Nutrients. 2018 Nov 23;10(12):1822. doi: 10.3390/nu10121822 (PMC6315936; doi:10.3390/nu10121822)
Supplement: Supplementary file 1 [file nutrients-10-01822-s001.pdf]

Supplementary Table S1: A typical daily menu in the 7-day diet.

**A typical daily menu**		
Breakfast	Oatmeal with apple/raisins or focaccia	Mixed powder with juice
Lunch	Salad with kidney beans and different fruits and vegetables	Mixed powder with juice
Dinner	Vegetable casserole/vegetable soup/sweet potato casserole/bean salad	Mixed powder with juice
Snack	Brazil nuts / fruits	Mixed powder with juice
5 capsules of n-3 supplements in the PUFA-Cys/Met diet		
2 capsules of n-3 supplements in the SFA+Cys/Met diet		

Supplementary Table S2: Average nutrient content and composition in the 7-day diet for each intervention group.

Nutrient content	Cys/Met_{low} + PUFA		Cys/Met_{high} + SFA	
	**Women**	**Men**	**Women**	**Men**
Kcal	1987.3	2467.6	1995.7	2504.7
Total fat, *% of energy*	32.0	31.9	31.9	32.6
Total fat, *g*	70.6	87.3	71.4	91.3
SFA, *% of energy*	5.5	5.2	13.5	13.3
SFA, *g*	12.4	14.6	30.3	37.2
PUFA, *% of energy*	10.9	10.6	3.3	3.2
PUFA, *g*	22.9	29.0	7.4	9.0
n-3 PUFA, *g*	5.2	5.9	1.5	1.8
n-6 PUFA, *g*	18.3	22.1	5.0	6.2
MUFA, *% of energy*	12.5	13.2	11.8	12.9
MUFA, *g*	27.6	36.2	26.5	36.0
Carbohydrates, *% of energy*	52.7	51.7	52.0	50.7
Carbohydrates, *g*	262.1	319.5	262.1	319.6
Protein, *% of energy*	12.2	12.7	13.1	13.3
Protein, *g*	60.6	78.2	66.0	83.7
Sulfur amino acids, *g*	1.0	1.2	5.8	6.0
Methionine, *g*	0.5	0.6	2.1	2.2
Cysteine, *g*	0.5	0.6	3.7	3.8

Data are expressed as means. Cys/Met_{low}+PUFA, diet low in cysteine and methionine and high in polyunsaturated fatty acids; Cys/Met_{high}+SFA, diet high in cysteine, methionine and saturated fatty acids; SFA, saturated fatty acids; PUFA, polyunsaturated fatty acids; MUFA, monounsaturated fatty acids

Supplemental Table S3: Change from baseline in metabolic parameters

	Cys/Met_{low} + PUFA		Cys/Met_{high} + SFA		*p*
	**Baseline**	**End of study**	**Baseline**	**End of study**	
Weight (kg)	66.8 (65.5 - 68.4)	66.4 (64.0 - 67.2)	65.7 (62.9 - 71.5)	64.6 (62.2 - 71.6)	0.37
BMI (kg/m^2)	22.6 (22.3 - 23.4)	22.4 (21.8 - 22.8)	22.3 (21.7 - 23.5)	22.3 (21.8 - 23.1)	0.32
Body fat percent	27.5 (20.1 - 28.3)	26.4 (19.7 - 28.2)	26.0 (20.5 - 28.8)	25.2 (19.5 - 27.1)	0.62
Waist circumference (cm)	75.0 (70.5 - 80.3)	76.0 (70.0 - 79.0)	75.0 (71.0 - 77.5)	73.5 (69.0 - 75.8)	0.65
Hip circumference (cm)	101.0 (98.3 - 101.5)	100.0 (96.3 - 101.8)	99.0 (96.0 - 100.8)	98.0 (95.5 - 101.0)	0.90
Waist-hip ratio	0.74 (0.71 - 0.80)	0.74 (0.72 - 0.78)	0.75 (0.73 - 0.78)	0.75 (0.71 - 0.76)	0.90
Systolic blood pressure (mm/Hg)	110 (102 - 119)	103 (100 - 108)	106 (101 - 112)	105 (100 - 109)	0.06
Diastolic blood pressure (mm/Hg)	70 (68 - 73)	64 (60 - 67)	65 (61 - 67)	59 (58 - 62)	0.12
Heart rate (bpm)	64 (63 - 65)	62 (55 - 65)	60 (56 - 73)	63 (55 - 70)	0.90
Mean arterial pressure (mm/Hg)	82 (80 - 94)	79 (76 - 83)	80 (77 - 83)	76 (75 - 78)	0.12
Glucose 0 h (mmol/L)	4.5 (4.4 - 5.2)	5.0 (4.8 - 5.3)	4.5 (4.1 - 5.0)	5.0 (4.7 - 5.2)	0.90
Glucose 2 h (mmol/L)	5.0 (4.4 - 5.9)	5.5 (5.4 - 6.2)	6.4 (6.4 - 6.9)	5.9 (4.9 - 6.6)	0.14
Total cholesterol (mmol/L)	4.4 (4.3 - 4.5)	3.8 (3.6 - 3.9)	3.8 (3.6 - 4.0)	3.9 (3.6 - 4.0)	0.055
HDL cholesterol (mmol/L)	1.6 (1.4 - 1.9)	1.4 (1.3 - 1.8)	1.5 (1.4 - 1.9)	1.3 (1.3 - 1.7)	0.95
LDL cholesterol (mmol/L)	2.5 (2.3 - 2.6)	2.0 (1.9 - 2.4)	2.0 (1.8 - 2.1)	2.1 (1.9 - 2.2)	0.095
Triglycerides (mmol/L)	0.7 (0.6 - 0.9)	0.5 (0.5 - 0.6)	0.8 (0.6 - 1.0)	0.6 (0.6 - 0.8)	0.092

Data are expressed as median (25 - 75th percentile) at baseline and end of study
p-values between groups median change was obtained by Wilcoxon signed-rank test
Cys/Met_{low}+PUFA, diet low in cysteine and methionine and high in polyunsaturated fatty acids;
Cys/Met_{high}+SFA, diet high in cysteine, methionine and saturated fatty acids; BMI, body mass index,
HDL, high-density lipoprotein; LDL, low-density lipoprotein

Supplementary Table S4: Fatty acids and SCD activity indices.

Fatty acid	Group	Baseline	Day 1	Day 7	*p* for interaction
C16	Cys/Met_{low}+PUFA	1710 (1410, 2010)	1690 (1380, 2010)	1460 (1140, 1770)	0.200
	Cys/Met_{high}+SFA	1470 (1170, 1780)	1560 (1250, 1860)	1510 (1210, 1820)	
C16:1	Cys/Met_{low}+PUFA	180 (122, 239)	208 (146, 270)	151 (88.5, 213)	0.700
	Cys/Met_{high}+SFA	164 (106, 223)	155 (95.5, 215)	168 (108, 227)	
C18	Cys/Met_{low}+PUFA	618 (547, 688)	546 (471, 622)	440 (365, 516)	0.055
	Cys/Met_{high}+SFA	520 (449, 591)	519 (447, 592)	507 (434, 579)	
C18:1	Cys/Met_{low}+PUFA	1720 (1420, 2010)	1600 (1300, 1910)	1370 (1070, 1680)	0.150
	Cys/Met_{high}+SFA	1510 (1220, 1800)	1460 (1160, 1760)	1470 (1170, 1770)	
SCD-16	Cys/Met_{low}+PUFA	0.101 (0.085, 0.12)	0.111 (0.094, 0.13)	0.0993 (0.082, 0.12)	0.650
	Cys/Met_{high}+SFA	0.109 (0.092, 0.13)	0.0982 (0.082, 0.12)	0.109 (0.092, 0.13)	
SCD-18	Cys/Met_{low}+PUFA	2.82 (2.51, 3.12)	2.91 (2.59, 3.23)	3.11 (2.79, 3.43)	0.500
	Cys/Met_{high}+SFA	2.86 (2.55, 3.16)	2.78 (2.47, 3.09)	2.85 (2.54, 3.16)	

Estimated marginal mean (95% CI) fatty acid concentrations at baseline, day 3 and day 7 in diet interventions. *p*-values are computed using linear mixed model regression with group, time and group$^{\times}$time interaction term in the model. Cys/Met_{low}+PUFA, diet low in cysteine and methionine and high in polyunsaturated fatty acids; Cys/Met_{high}+SFA, diet high in cysteine, methionine and saturated fatty acids; SCD, stearoyl-CoA desaturase
